# Novel exopolysaccharide derived from probiotic *Lactobacillus pantheris* TCP102 strain with immune-enhancing and anticancer activities

**DOI:** 10.3389/fmicb.2022.1015270

**Published:** 2022-09-26

**Authors:** Shouxin Sheng, Yubing Fu, Na Pan, Haochi Zhang, Lei Xiu, Yanchen Liang, Yang Liu, Bohui Liu, Cheng Ma, Ruiping Du, Xiao Wang

**Affiliations:** ^1^State Key Laboratory of Reproductive Regulation & Breeding of Grassland Livestock, School of Life Sciences, Inner Mongolia University, Hohhot, China; ^2^School of Life Sciences, Faculty of Medicine and Life Sciences, State Key Laboratory of Cellular Stress Biology, Xiamen University, Xiamen, China; ^3^Animal Nutrition Institute, Agriculture and Animal Husbandry Academy of Inner Mongolia, Hohhot, China

**Keywords:** exopolysaccharide, probiotic, *Lactobacillus pantheris*, immune-enhancing activity, anticancer activity

## Abstract

Probiotics are gaining attention due to their functions of regulating the intestinal barrier and promoting human health. The production of exopolysaccharide (EPS) is one of the important factors for probiotics to exert beneficial properties. This study aimed to screen exopolysaccharides-producing lactic acid bacteria (LAB) and evaluate the probiotic potential. we obtained three exopolysaccharide fractions (EPS1, EPS2, and EPS3) from *Lactobacillus pantheris* TCP102 and purified by a combination of ion-exchange chromatography and gel permeation chromatography. The structures of the fractions were characterized by FT-IR, UV, HPLC, and scanning electron microscopy (SEM) analysis. The Mw of EPS1, EPS2, and EPS3 were approximately 20.3, 23.0, and 19.3 kDa, and were mainly composed of galactose, glucose, and mannose, with approximate molar ratios of 2.86:1:1.48, 1.26:1:1, 1.58:1.80:1, respectively. Furthermore, SEM analysis demonstrated that the three polysaccharide fractions differ in microstructure and surface morphology. Additionally, preliminary results for immune-enhancing and anticancer activities reveal that these EPSs significantly induced the production of nitric oxide (NO), TNF-α, and IL-6 in Ana-1 cells and peritoneal macrophage cells. Meanwhile, the EPSs also significantly suppressed the proliferation of HCT-116, BCG-803, and particularly A-2780 cells. The results suggest that the three novel EPSs isolated from *Lactobacillus pantheris* TCP102 can be regarded as potential application value in functional food and natural antitumor drugs.

## Introduction

Probiotics, which the WHO defines as “live microorganisms when administered in adequate quantities confer a health profit to the host cell,” play important roles in preventing disease and promoting health of the host (Ouwehand, 2017). Most probiotics are bacteria belonging mainly to the genera *Lactobacillus* and *Bifidobacterium*. Probiotics have been shown to be effective in mitigating gastrointestinal inflammatory conditions such as inflammatory bowel disease, irritable bowel syndrome, certain respiratory conditions, and allergies (Quigley, 2013; Butel, 2014; Plaza-Diaz et al., 2014). In addition to regulating intestinal epithelial homeostasis and immune responses, certain probiotics have been reported to have anticancer activity through different mechanisms (Chong, 2014; Thomas et al., 2014; Kayama and Takeda, 2016). Furthermore, research indicates that certain probiotic functions are related to bacterial secretions (Shida et al., 2011).

Various genera of lactic acid bacteria (LAB) are capable of synthesizing exopolysaccharide (EPS) that are either attached to the cell surface or are found in the extracellular medium as slime (Nwodo et al., 2012; Patel et al., 2012; Zannini et al., 2016). EPS is widely used in the biotechnology, health, and pharmaceutical industries and is widely accepted as food products. Many reports have indicated that EPS has various potential physiological functions and biological activities. For example, various EPSs are known to have antioxidant, anticancer, immune-enhancing, antibacterial, hypoglycemic, and antihypertensive activities (Sengül et al., 2011; Nácher-Vázquez et al., 2015; El-Deeb et al., 2018). The therapeutic activity of polysaccharides depends on their structural features such as molecular weight, chemical structure, and the conformation and configuration of the glycosidic linkages. These features, in turn, are dependent on various biotic and abiotic factors such as biomass morphology, pH, oxygen, fermentation time, temperature, stirring, carbon and nitrogen contents, and the C/N ratio of the culture medium (Champagne et al., 2006; Ruhmkorf et al., 2012; Ma et al., 2014). The structure-function relationship of EPS remains a major research topic (Surayot et al., 2014; Gentès et al., 2015).

An increasing number of researchers are investigating the use of EPS with macrophage immune-enhancing activity. Macrophages are an important part of the innate immune system. They interact and coordinate with adaptive immune responses through the production of cytokines and phagocytosis of pathogens. A hallmark of macrophage activation is the release of cytokines and NO. For example, microbial exopolysaccharides can increase the activities of IL-2 in the supernatant of murine splenocytes and IL-6 and TNF-α in the supernatant of murine macrophages, by promoting the expression of the mRNAs of these cytokines (Chen et al., 2006; Ren et al., 2016; Wang et al., 2016). In addition, EPS has been reported to exhibit intense antitumor activities. EPS treatment observably altered the cell morphology and even damaged the human cancer cell lines HepG-2, BGC-823, and HT-29, and could induce cancer cell apoptosis (Chen et al., 2013; Li et al., 2014b,^2015^).

In the present study, we evaluated the probiotic potential of 18 strains of lactic acid bacteria isolated from tomato pomace in our laboratory. As basic properties of probiotics, acid resistance, bile salt tolerance, and adhesion ability were investigated. We isolated three different polysaccharides named EPS1, EPS2, and EPS3 from a TCP102 culture. The chemical structure and physicochemical properties of the purified EPSs were characterized by high performance liquid chromatography (HPLC), ultraviolet (UV) and Fourier transform infrared spectroscopy (FT-IR), and scanning electron microscopy (SEM). This research aimed to isolate lactic acid bacteria from tomato pomace to assess their potential probiotic properties and provide information on the structure-function relationship between the characteristics of the EPSs and their potential bioactivities, such as immune-enhancing activity in murine macrophages and antiproliferative effects with three human cancer cells. This study will provide the scientific reference for further systematic investigation and application in immune-enhancing functional food and natural antitumor drugs of lactic acid bacteria polysaccharides.

## Materials and methods

### Strains and culture condition

Eighteen strains of lactic acid bacteria were isolated from tomato pomace by our laboratory. The strains were maintained at −80°C in MRS broth (Guangdong Huankai, China) containing 30% (v/v) glycerinum (Sinopharm Chemical Reagent Co., Ltd, China). The strain was propagated successively in MRS agar then in MRS broth at 37°C for 18 h, prior to the experiments. A batch fermentation was performed for 30 h at 37°C under anaerobic conditions. All strains were identified based on cell morphology, biophysical and biochemical tests, and 16S rRNA gene sequence analysis. Strains TCP001, TCP004, TCP007, TCP008, TCP009, TCP015, TCP016, TCP017, TCP024, TCP029, TCP037, TCP045, TCP050, TCP063, TCP071, TCP073, TCP080, and TCP102 were unambiguously identified as *Lactobacillus harbinensis*, *Lactobacillus paracasei subsp. paracasei*, *Lactobacillus fermentum*, *Lactobacillus plantarum*, *Lactobacillus rhamnosus*, *Lactobacillus coryniformis subsp. torquens*, *Lactobacillus buchneri*, *Lactobacillus manihotivorans*, *Lactobacillus parafarraginis*, *Lactobacillus camelliae*, *Lactobacillus rapi*, *Lactobacillus pontis*, *Lactobacillus helveticus*, *Lactobacillus vaginalis*, *Lactobacillus amylovorus*, *Lactobacillus hilgardii*, *Lactobacillus panis*, and *Lactobacillus pantheris*.

The indicator bacteria of antibacterial experiment were *Escherichia coli* CMCC 44102 and *Staphylococcus aureus* ATCC 27543, which were purchased from Beijing Nanolink Biotechnology Research Institute and American Culture Collection Center. The indicator strains were cultivated in LB broth (Guangdong Huankai, China) with shaking at 37°C for 24 h.

### The evaluation of probiotic properties of lactic acid bacteria

#### Bile salt tolerance

The ability of isolates to grow in presence of bile salt was measured. This was carried out with 0.3% w/v oxgall, 0.5% w/v oxgall, 1.0% w/v oxgall and control was maintained employing MRS broth. The samples were inoculated at 37°C for 24 h and OD_600_ of samples was measured to check the viability of cells. The oxgall resistance was determined by the following equation: Survival Rate (%): [OD (After treatment)/OD (Before treatment)] × 100%.

#### Gastric tolerance

The resistance of strains to gastric was measured as described by Wang et al. (2021), with minor modifications. Lactic acid bacteria that were frozen at −80°C were activated and inoculated into MRS broth. Take 5 ml of bacterial liquid and centrifuge at 3,000 rpm for 10 min to discard the supernatant. Add 5 ml of sterile saline and mix to make the bacterial suspension. Take 1 ml of bacterial suspension and mix with 9 ml of artificial gastric juice, respectively. The samples were inoculated at 37°C for 0 h and 3 h, and calculate the survival rate by serial dilution and plating, three replicates for each strain. The survival rate was calculated using the following equation: Survival Rate (%): [OD (After treatment)/OD (Before treatment)] × 100%.

#### Adhesion to Caco-2 cells

The ability of the tested bacteria to adhere to the Caco-2 cell layer was investigated according to the previously published method (Sałański et al., 2022) with minor modifications. For this purpose, Caco-2 cells were cultured in RPMI medium supplemented with 10% heat-inactivated fetal bovine serum and 1% penicillin–streptomycin blend and cells were refined on 24-well tissue culture plates and incubated at 37°C in 5% CO_2_ under a relatively humidified atmosphere until a confluent monolayer was formed. Sometime recently the attachment measure, the media within the wells containing a Caco-2 cell monolayer were evacuated and supplanted with new antibiotic-free RPMI. From that point, 1 × 10^8^ CFU/mL of isolates was included to each well with a add up to volume of 1 mL and after that incubated for 3 h at 37°C under an atmosphere of 5% (v/v) CO_2_. The wells were washed twice with a sterile pre-warmed PBS solution to evacuate non-attached bacterial cells. 1 mL of 1% (v/v) Triton X-100 was included to each well to withdraw the cells from the wells and the blend was mixed for 10 min. To measure the viable cell count, the cell suspension was plated onto MRS agar and hatched at 37°C. Each experiment was made at least in three independent biological replicates with triplicate technical repetitions.

#### Antimicrobial activity

The antimicrobial features of isolates were performed by agar well diffusion assay.

In this method, plates containing *Escherichia coli* and *Staphylococcus aureus* agar medium impregnated with different indicator bacteria was used. The oxford cup was used to create wells in medium. Finally, 100 μL supernatants of isolates were placed inside each well and plates were then incubated at 37°C for 24 h. After incubation time, the measured inhibition halo zone diameters were statistically analyzed.

### Extraction, isolation, and purification of exopolysaccharide

Culture medium of lactic acid bacteria was heated in boiling water for 10 min to inactivate enzymes, cooled down to 25°C centrifuged (20 min, 5,500 rpm, 4°C) to remove cells and coagulated proteins, and then the supernatant was collected. Trichloroacetic acid (80%, w/v) was added to the supernatant to a final concentration of 4% (w/v) with gentle stirring and the solution was kept at 4°C for 10 h. The precipitated proteins were removed by centrifugation (15 min, 10,000 rpm, 4°C), and the supernatant was precipitated by slowly adding four volumes of prechilled absolute ethyl alcohol and incubating at 4°C overnight (Ai et al., 2008; Wang et al., 2014). The EPS was collected by centrifugation at the same conditions as described above (15 min, 10,000 rpm, 4°C). The precipitates were dialyzed (Mw cut-off: 8,000-14,000 Da) with three changes of water per day for 3 days. The dialysate was then lyophilized, and collected as crude EPS.

The crude EPS was purified by a two-step process. Samples were first applied to a DEAE-Sepharose Fast Flow (GE, USA) ion exchange column (26 mm × 400 mm), and eluted with Tris-HCl buffer (55 mM, pH 7.5–7.8), followed by and a linear gradient of 0–1 M NaCl buffer. The eluate fractions were collected at a rate of 6 mL per tube using a fraction collector (Ai et al., 2008). The yield and total sugar content of EPS were measured by the phenol-sulfuric acid method using 0–200 mg/L of glucose as a standard (Matsuzaki et al., 2015). One polysaccharide fraction named EPS1 was obtained during the elution using Tris–HCl buffer, while another two fractions, named EPS2 and EPS3, were eluted using the linear gradient of NaCl. EPS fractions were dialyzed against deionized water with three changes of water per day for 3 days at 4°C, and then lyophilized. The three fractions were further purified on a Sepharose CL-6B (GE, USA) gel column (16 mm × 1,000 mm). Each fraction was eluted with 55 mM Tris–HCl buffer (pH 7.6–7.8) at a flow rate of 0.5 mL/min, collecting 6 mL of eluate per tube. The eluates with EPS were detected, collected, dialyzed against deionized water with three changes of water every day for 3 days at 4°C. The dialysates were lyophilized, and the resulting products consisted of the major purified fractions that were used for further study. The total carbohydrate content of each fraction was measured using the phenol-sulfuric acid method with glucose as standard (Matsuzaki et al., 2015).

### Estimation of homogeneity and apparent molecular weight

To estimate the homogeneity and apparent molecular weight of purified TCP102 EPSs, samples were separated on a BRT105-104-102 (Shimadzu) tandem gel column (8 mm × 300 mm). The samples were eluted isostatically with ultrapure water (vacuum-filtered through 0.22 μm membrane filter and degassed), at 40°C and a flow rate of 0.8 mL/min. Aqueous standards and EPS fractions (5 mg/mL and 10 μL, respectively) were filtered through 0.22 μm cellulose acetate filters (Análisis Vínicos, Tomelloso, Toledo, Spain) and injected (20 μL) into the HPLC. The system was calibrated with glucan molecular weight standards (1,152, 11,600, 23,800, 48,600, 80,900, 148,000, 273,000, and 409,800 Da). Peaks were detected using a refractive index detector (RI) (Waters).

### Monosaccharide composition analysis

To determine the monosaccharide composition of purified TCP102 EPS, a 10 mg sample was hydrolyzed with 4 mL of 4 mol/L trifluoroacetic acid (TFA) at 120°C for 2 h under a N_2_ environment. The hydrolysates were dried under a stream of N_2_ and dissolved with 10 mL ultrapure water. The IC (ion chromatography) system used was a Dionex ICS3000 equipped with a conductivity detector and a Carbo PacTMPA20 analytical column (3 mm × 150 mm) and running at a flow rate of 0.5 mL/min at 35°C. Peaks were assigned based on peak retention times of a standard. The diluted samples were filtered through a 0.22 μm syringe filter prior to injection.

### Ultraviolet and Fourier transform infrared spectroscopy spectrometric analysis

Ultraviolet–visible (UV–vis) spectroscopy was combined with HPLC (GE, USA). The EPS solution was prepared for HPLC by suspending a sample in distilled water to a concentration of 500 μg/mL. Spectroscopy was conducted within the wavelength range of 190–400 nm.

The GPC system included a surveyor pump, Surveyor Autosampler (Themo Electron Corporation, Waltham, MA, USA), a Surveyor Photodiode Array Detector, two columns (Ultrahydrogel 500 and Ultrahydrogel 250 column, 7.8 mm × 300 mm) connected serially. The mobile phase was an isocratic solvent system consisting of acetic acid and sodium acetate (sodium acetate 18 g, acetic acid 9.8 mL, plus ultrapure water to 1 L) running at a flow rate of 0.9 mL/min. The temperature of column was 45°C. Solutions were filtered through a 0.22 μm filter and degassed prior to use.

The various functional groups of EPS fractions were determined using a Fourier transform infrared spectrophotometer (Thorlabs Inc, Newton, NJ, USA). The purified EPS samples (1–2 mg) were ground with 100–200 mg KBr powder and pressed into pellets prior to FTIR measurements in the frequency range of 4,000–400 cm^–1^.

### Scanning electron microscopy analysis

The microstructure and surface morphology of the three purified EPS fractions were investigated using an S-4800 scanning electron microscope (Hitachi, Japan) at an accelerating voltage of 10.0 kV. Purified EPS samples were glued onto SEM stubs and gold-coated before analysis.

### Immune-enhancing activity of exopolysaccharide

The mouse macrophage cell line Ana-1 was purchased from the Type Culture Collection of the Chinese Academy of Sciences (Shanghai, China). Peritoneal macrophage cells were obtained from the lateral wall of the peritoneum of Ana-1 mice. Cells were cultured in RPMI-1640 medium (GE Healthcare Life Sciences, Hy Clone Laboratories, Utah, USA) containing 100 μg/mL penicillin, 100 μg/mL streptomycin, 2 mM L-glutamine, and 10% fetal bovine serum (FBS; GE Hy Clone). The 75-cm^2^ culture flasks were incubated at 37°C in a 5% CO_2_ atmosphere. The cell suspension was then pipetted into the wells of a 96-well plate at the rate of 200 μL/well (2 × 10^5^ cells/mL). Cells were preincubated for 4 h, then treated with 200 μL of different concentrations of EPS (0, 31.25, 62.5, 125, 250, and 500 μg/mL) suspended in RPMI-1640 medium for 24 h. LPS (1 μg/mL) was used as the positive control.

NO production was measured using a Griess reagent kit (Promega Corporation, Beijing, China) following the manufacturer’s protocol. The absorbance was monitored at 540 nm with a microplate reader (Thermo Fisher Scientific, Shanghai, China). Following NO measurements, the culture supernatants of macrophage cells were collected to estimate the amount of secreted TNF-α and IL-6 reagent (R&D Systems, Minneapolis, USA) using ELISA.

### Anticancer activity

The human colon cancer, ovarian cancer, and gastric cancer cell lines HCT-116, A-2780, and BCG-803, respectively (all cancer cell lines were purchased from the ATCC, Manassas, VA, USA), were cultured using DMEM (GE, Hy Clone) containing 100 μg/mL penicillin, 100 μg/mL streptomycin, 2 mM L-glutamine, and 10% FBS in 75-cm^2^ culture flasks at 37°C under a 5% CO_2_ atmosphere. To measure the inhibitory rates of EPS on the growth of cancer cells, the cell suspensions were pipetted into a 96-well plate at the rate of 200 μL/well (2 × 10^5^ cells/mL). After 4 hours, the cancer cells were treated with 200 μL of various concentrations of purified EPS (0, 31.25, 62.5, 125, 250, and 500 μg/mL) and 5-FU (5-fluorouracil, 50 μg/mL) for 24, 48, and 72 h. At the end of each treatment, MTS reagent (Promega Corporation, Beijing, China) was added to each well (20 μL/well), and the plates were incubated at 37°C for 2 hours in a humidified, 5% CO_2_ atmosphere. The absorbance at 490 nm was measured using a 96-well plate reader. The inhibitory rate was calculated using the following equation:


InhibitoryRate(%)

 =[1-(A-s⁢a⁢m⁢p⁢l⁢eA)b⁢l⁢a⁢n⁢k/(A-c⁢o⁢n⁢t⁢r⁢o⁢lA)b⁢l⁢a⁢n⁢k]×100%

where A_*sample*_ is the absorbance of the reagent mixture with EPS or 5-FU, A_*blank*_ is the absorbance of the reagent mixture with medium, and A_*control*_ is the absorbance of the reagent mixture without EPS and 5-FU.

### Statistical analysis

All experiments were repeated thrice. Results were expressed as the mean ± SD of triplicate analyses. Statistical significance was analyzed by one-way ANOVA using SPSS16.0 software. Asterisks indicate the *P* values of the comparison between treatment means versus the control group, where *, ^**^, and ^***^ indicate *P* < 0.05, *P* < 0.01, and *P* < 0.001, respectively.

## Results

### Identification of strains

The 16S-rRNA gene sequencing was used to validate the phenotypic characterization of the selected lactic acid bacteria isolates. The PCR-amplified 1,544 bp fragments of the 16S-rRNA gene of the isolates were sequenced and blasted with the sequences deposited in GenBank. Amplification of the 16S-rRNA genes of 18 LAB isolates confirmed that all of which belonged to the genus *Lactobacillus*, which had been identified in our previous study. In addition, the nucleotide sequences of the 16S rRNA gene had been deposited with GenBank (Wu et al., 2014).

### Acid resistance

**FIGURE 1:**
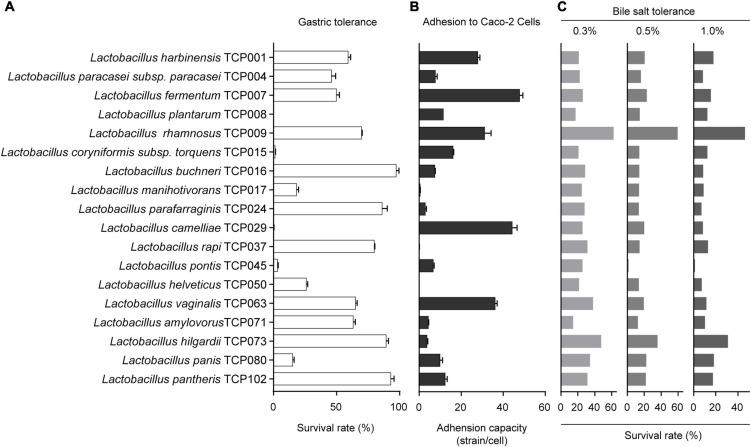
The evaluation of probiotic properties of lactic acid bacteria. **(A)** The results of the tolerance of 18 lactic acid bacteria strains to artificial gastric juice. **(B)** The adhesion results of 18 lactic acid bacteria strains to Caco-2 cells. **(C)** The results of bile salts tolerance of 18 lactic acid bacteria strains.

The results of the tolerance of 18 lactic acid bacteria strains to artificial gastric juice are shown in Figure 1A. The strains of TCP015, TCP029, and TCP045 could hardly survive in gastric juice, and the survival rate of strains of TCP016, TCP024, TCP073, TCP102, and TCP037 in artificial gastric juice was greater than 80%. The survival rate of TCP001, TCP007, TCP071, TCP063, and TCP009 was between 50 and 80%, in addition, the survival rate of TCP008 and TCP050 was between 20 and 50%, and the survival rate of TCP016, TCP017 and TCP080 was between 10 and 20%. The results showed that the survival rate of most lactic acid bacteria in gastric juice was above 10%, which could tolerate the environment of gastric acid.

### Adhesion assay

The adhesion results of 18 lactic acid bacteria strains to Caco-2 cells are shown in Figure 1B. The results showed that the adhesion ability of different lactic acid bacteria to Caco-2 cells was very different, among which TCP017, TCP050, and TCP037 had almost no adhesion ability. The strains of TCP004, TCP016, TCP024, TCP045, TCP071, and TCP073 adhered to an average of 3-10 bacteria per cell, and the strains of TCP008, TCP015, TCP102, and TCP080 adhered 10-20 bacteria per cell. In addition, the five strains of TCP001, TCP007, TCP029, TCP063, and TCP009 had strong adhesion ability, with an average of 28–48 bacteria adhered to each cell.

### Bile salt tolerance

The survival rates of 18 isolates in 0.3, 0.5, and 1.0% oxgall are indicated in Figure 1C, and these bacteria have a certain tolerance to 0.3, 0.5, 1.0% oxgll, despite variations in the degree of viability. Among them, TCP009 had the highest tolerance to all concentrations of bile salts, and was significantly different from other strains. The five strains TCP073, TCP102, TCP080, TCP063, and TCP037 had a high tolerance to 0.3% bile salts, ranging from 30% to 50%, except for TCP008 and TCP071, which had a tolerance to 0.3% bile salts. Except for 10–20%, the rest of the strains were between 20% and 30%. The tolerance to 0.5 and 1.0% bile salts was the highest among TCP073 and TCP009. Except for TCP045, which was extremely low, the rest of the strains were almost between 10 and 20%.

### Antimicrobial activity

The bacteriostatic results of 18 strains against *Escherichia coli* and *Staphylococcus aureus* are shown in Table 1. The results showed that these 18 strains had stronger bacteriostatic effect on *Staphylococcus aureus* than *Escherichia coli*. TCP009, TCP008, TCP045, TCP071, TCP063, and TCP004 had strong antibacterial effects on *Escherichia coli.* In addition, TCP009, TCP004, TCP015, TCP017, TCP050, TCP073, and TCP080 had the strongest antibacterial effect on *Staphylococcus aureus*.

**TABLE 1:** The results of antibacterial activity for the strains against various pathogens.

Indicator bacteria	Zone diameter (mm)																	
	TCP 001	TCP 004	TCP 007	TCP 008	TCP 009	TCP 015	TCP 016	TCP 017	TCP 024	TCP 029	TCP 037	TCP 045	TCP 050	TCP 063	TCP 71	TCP 073	TCP 080	TCP 102
*Escherichia coli*	12.00 ± 0.20	14.00 ± 0.10	12.00 ± 0.22	16.00 ± 0.24	21.00 ± 0.10	12.40 ± 0.30	6.50 ± 0.10	11.80 ± 0.10	0.00 ± 0.00	0.00 ± 0.00	8.50 ± 0.10	17.40 ± 0.10	0.00 ± 0.00	17.40 ± 0.10	16.50 ± 0.10	13.50 ± 0.10	11.50 ± 0.10	5.40 ± 0.20
*Staphylococcus aureus*	18.30 ± 0.10	21.60 ± 0.10	15.50 ± 0.10	15.50 ± 0.10	21.60 ± 0.10	20.30 ± 0.20	19.60 ± 0.10	21.70 ± 0.10	16.40 ± 0.10	18.50 ± 0.10	19.60 ± 0.10	19.60 ± 0.10	24.40 ± 0.20	17.40 ± 0.20	17.60 ± 0.10	20.50 ± 0.10	20.70 ± 0.20	18.60 ± 0.10

### Extraction and purification of exopolysaccharide

**FIGURE 2:**
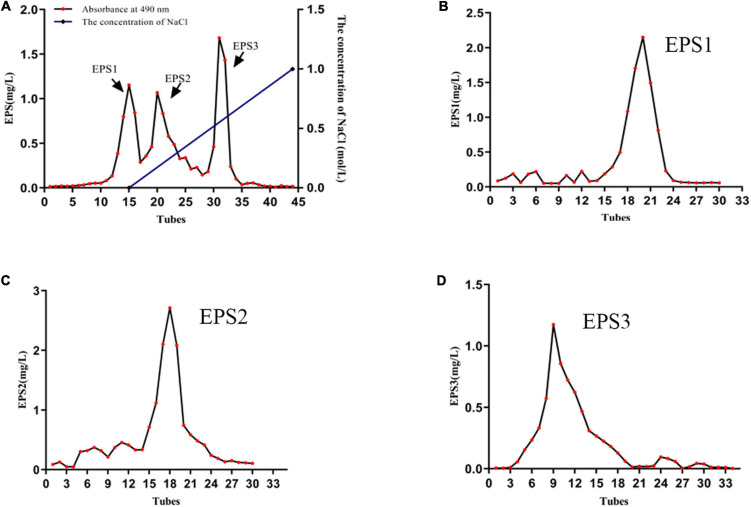
The characterization of EPSs. **(A)** Elution profile of EPSs produced by strain *L. pantheris* TCP102 on DEAE-Sepharose fast flow column. Elution profiles of **(B)** EPS1, **(C)** EPS2, and **(D)** EPS3 on Sepharose CL-6B gel columns.

The results showed that 11 lactic acid bacteria strains including TCP001, TCP004, TCP007, TCP008, TCP009, TCP016, TCP063, TCP071, TCP073, TCP080, and TCP102 had probiotic potential. Next, we selected the TCP102 strain with probiotic properties as a further study strain. Supplementary Figures 1A,B showed the smooth and whitish colony appearance of strain TCP102 on MRS agar. Colonies were round and medium-sized. The morphological characteristics and 16S rRNA gene sequence analysis identified strain TCP102 as *Lactobacillus pantheris*. The yield of crude exopolysaccharide was 525 mg/L based on analysis of MRS fermentation broth. Three homogeneous exopolysaccharides, designated EPS1, EPS2, and EPS3, were obtained after a two-step isolation and purification process. First, the crude EPS solution was separated through anion-exchange chromatography using DEAE-Sepharose Fast Flow. The resulting three elution peaks were detected by the phenol-sulfuric acid assay. EPS1 was eluted with Tris-HCl buffer, and was thus a neutral polysaccharide. EPS2 and EPS3 were eluted with Tris-HCl and a high ionic strength NaCl solution, which were acidic polysaccharides (Figure 2A). The three exopolysaccharides were purified using a Sepharose CL-6B gel column (Figures 2B–D), resulting in a single and relatively symmetrical peak on the elution profile for each fraction. The total sugar contents of EPS1, EPS2, and EPS3 were approximately 92.36, 95.75, and 93.24%, respectively, and all purified exopolysaccharides were colorless powders. Each of fractions were collected, dialyzed, and lyophilized for the following analysis.

### Molecular weight and monosaccharide composition of exopolysaccharide

**FIGURE 3:**
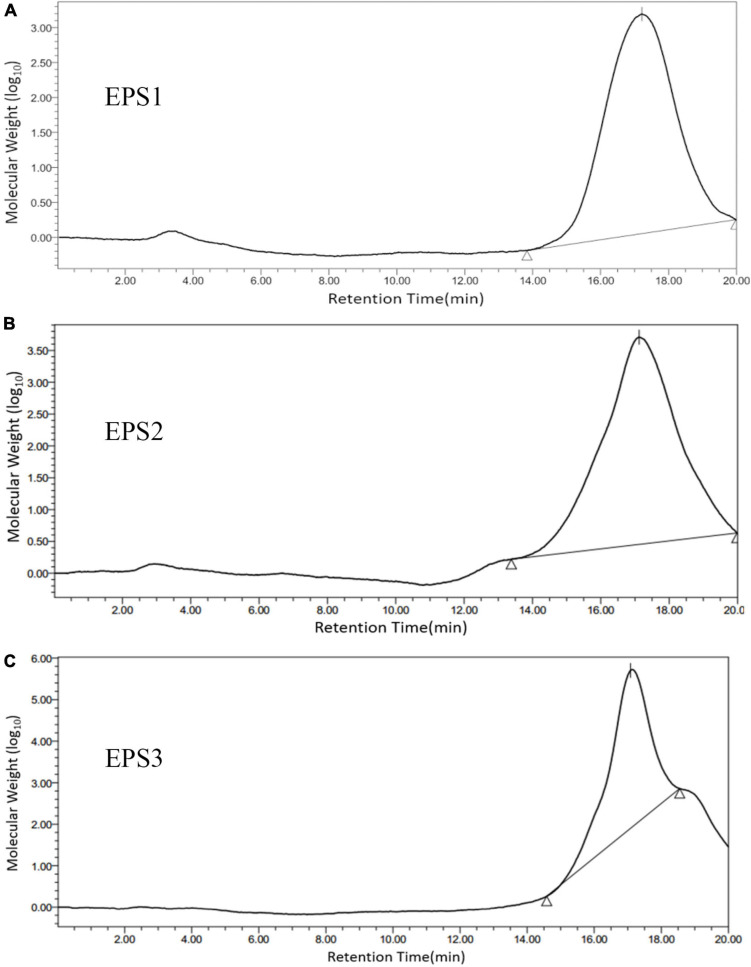
Molecular weight distribution of **(A)** EPS1, **(B)** EPS2, and **(C)** EPS3 on high performance gel permeation chromatogram (HPGPC). The samples were applied to a BRT105-104-102 system with a tandem gel column (8 mm × 300 mm, 8 μm) and eluted with 0.1 M ultrapure water at 0.8 mL/min and a column temperature of 40°C.

A GPC system was used to determine the purity and molecular weights (Mw) of the EPSs, that the chromatogram shows only one symmetrical peak corresponding to EPS1, EPS2, and EPS3 (Figure 3), indicating that the polysaccharides are homogeneous. The molecular weights of EPS1, EPS2, and EPS3 were estimated to be 20.3, 23.0, and 19.3 kDa, respectively, suggesting similar molecular weight distributions among the three purified polysaccharide fractions. The molecular weights of the three polysaccharides are lower than those of *Nitratireductor* sp. PRIM-31 (90 kDa) (Priyanka et al., 2016), *Tolypocladium* sp. fungus (40 kDa) (Yan et al., 2010), and *L. curvatus* DPPMA10 (100 kDa) EPSs (Minervini et al., 2010). In general, polysaccharides with lower molecular weights are more water soluble, with a relatively expanded chain conformation, which are associated with more bioactive molecules (Shi et al., 2016).

**FIGURE 4:**
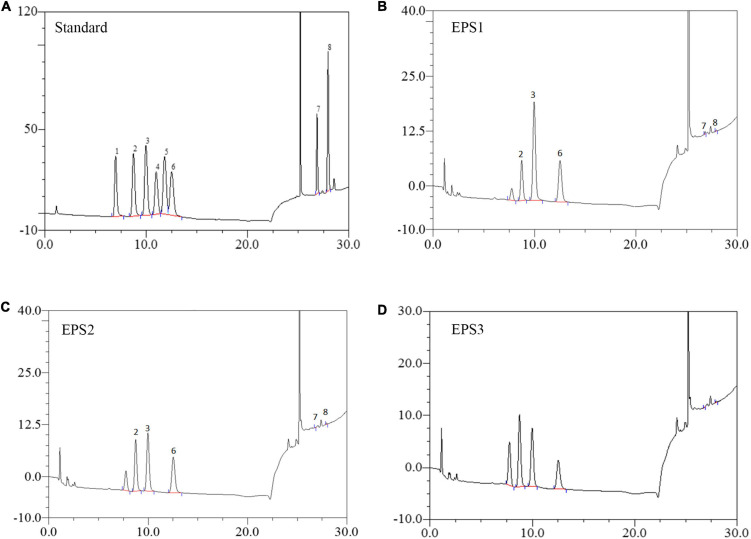
HPLC chromatogram of the monosaccharide components of EPSs from *L. pantheris* TCP102. **(A)** Monosaccharide standard, the peak values of 1–8 represent arabinose, galactose, glucose, xylose, mannose, ribose, galacturonic acid, and glucuronic acid, respectively. **(B)** EPS1, **(C)** EP2, and **(D)** EPS3.

Monosaccharide composition analysis (Figure 4) shows the presence mainly of galactose, glucose, and mannose with approximate molar ratios of 2.86:1:1.48, 1.26:1:1, and 1.58:1.80:1 in EPS1, EPS2, and EPS3, respectively. Furthermore, all the EPSs contain trace amounts of galacturonic acid and glucuronic acid. The analysis also yielded one unknown monosaccharide, which we designated as “n.a.”. Similar results were reported by Li et al., who found glucose, mannose, and galactose as the predominant components of the EPS of *Lactobacillus helveticus* MB2-1 (Li et al., 2014a). Moreover, glucose and mannose are also the primary components of the EPS of *Bifidobacterium animalis* RH and *Streptococcus thermophilus* 05–34 (Shang et al., 2013; Li et al., 2016).

### Ultraviolet and Fourier transform infrared spectroscopy analysis

**FIGURE 5:**
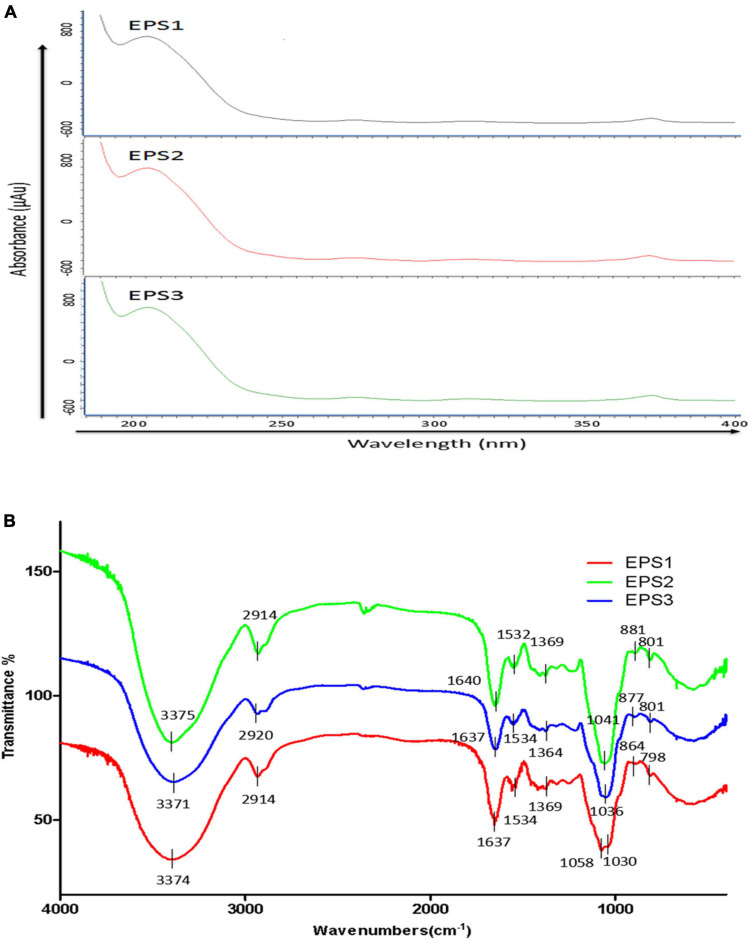
UV and FT-IR spectrometric analysis. **(A)** UV spectra of EPSs in the range of 180–540 nm. **(B)** FT-IR spectra of EPSs in the range of 600–4,000 cm^–1^.

An analysis of the ultraviolet scan spectrum of the EPSs is shown in Figure 5A. No absorption peaks were found at 260 and 280 nm, indicating an absence of proteins and nucleic acids. In the infrared wavelengths, the EPSs exhibit properties characteristic of polysaccharides. Specifically, the FT-IR spectrum (Figure 5B) shows common characteristic bands among EPS1, EPS2, and EPS3, the bands at 3,374, 3,375 and 3,371 cm^–1^ indicate the stretching vibration of the O-H group. The signals at 2,914, 2,914 and 2,920 cm^–1^ are attributed to the stretching vibration of the C-H bond (Liu et al., 2009). Meanwhile, the stronger absorption signals at 1,637, 1,640, and 1,637 cm^–1^ in the FT-IR spectra of EPSs indicate the dominance of the C = O stretching group (Shuhong et al., 2014). All above mentioned characteristic bonds are typical groups of polysaccharides. Furthermore, the peaks at 2,850 cm^–1^ are usually defined as the N-H bending of amides, and it couldn’t be found among EPS1, EPS2, and EPS3 in Figure 5B, which confirms that the protein was basically eliminated during the purification process. This statement is consistent with the results of UV spectrum in Figure 5A. EPS-1, EPS-2 and EPS-3 had no absorption peak near 1,730 cm^–1^, indicating that they were neutral polysaccharides. Moreover, the strong absorption signals at 1,058, 1,030, 1,041, and 1,036 cm^–1^ indicate the dominance of the pyran rings, especially signals at 1,058 cm^–1^ could be attributed to the α-(1→6) glycosidic bond in the main chain (Wu et al., 2009; Shuhong et al., 2014). The absorption signals at 864, 881, and 877 cm^–1^ in the IR spectra are characteristic of the β-anomeric configuration. In addition, the strong absorption signals at 798, 801, and 801 cm^–1^ indicate the presence of the α-anomeric configuration of mannose units, which agree with the results of monosaccharide composition of EPS (Mathlouthi and Koenig, 1987). These results suggest that all the three EPSs may be neutral polysaccharide with both α- and β-anomeric configuration, with the existence of α-(1→6) glycosidic bond.

### Scanning electron microscopy analysis

**FIGURE 6:**
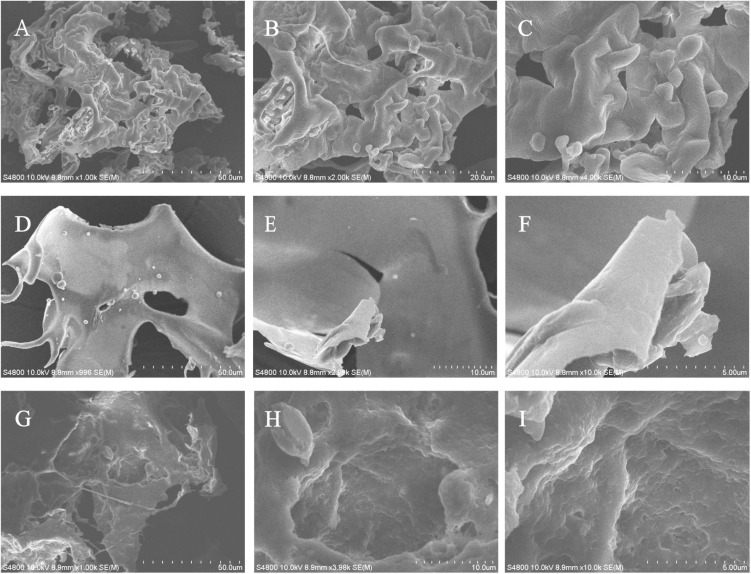
The microstructure and surface morphology of purified EPS fractions observed by SEM analysis. **(A)** SEM image of EPS1 in the scale bar of 50 μm. **(B)** SEM image of EPS1 in the scale bar of 10 μm. **(C)** SEM image of EPS1 in the scale bar of 5 μm. **(D)** SEM image of EPS2 in the scale bar of 50 μm. **(E)** SEM image of EPS2 in the scale bar of 10 μm. **(F)** SEM image of EPS2 in the scale bar of 5 μm. **(G)** SEM image of EPS3 in the scale bar of 50 μm. **(H)** SEM image of EPS3 in the scale bar of 10 μm. **(I)** SEM image of EPS3 in the scale bar of 5 μm.

Scanning electron microscopy has been widely used to characterize the surface morphology and the microstructure of polysaccharides. SEM images of EPS1, EPS2, and EPS3 are shown in Figure 6. EPS1 (Figure 6A) appears as a smooth sheet structure with many homogeneous rod-shaped lumps. Meanwhile, EPS2 (Figure 6B) is characterized by flakes piling over a compact structure with a rough surface. Lastly, EPS3 (Figure 6C) displays inner filaments composed of irregularly shaped particles, which is associated with greater viscosity, film forming properties, and water retention performance. In *L. plantarum* KX041, SEM micrographs of EPS shows an irregular, highly porous web-like structure, and an uneven surface (Xu et al., 2019). The EPS from *Lactobacillus pentosus* LZ-R-17 also displays a web-like structure, but its smooth surface probably improves its viscosity and water holding capacity (You et al., 2020). It should be noted that while EPS composition and structure can determine the its microstructure and surface morphology, many difference are most likely due to the different methods of sample extraction, preparation, and purification (Kanamarlapudi and Muddada, 2017).

### Immune-enhancing effect

#### Effect of exopolysaccharide on the production of nitric oxide

**FIGURE 7:**
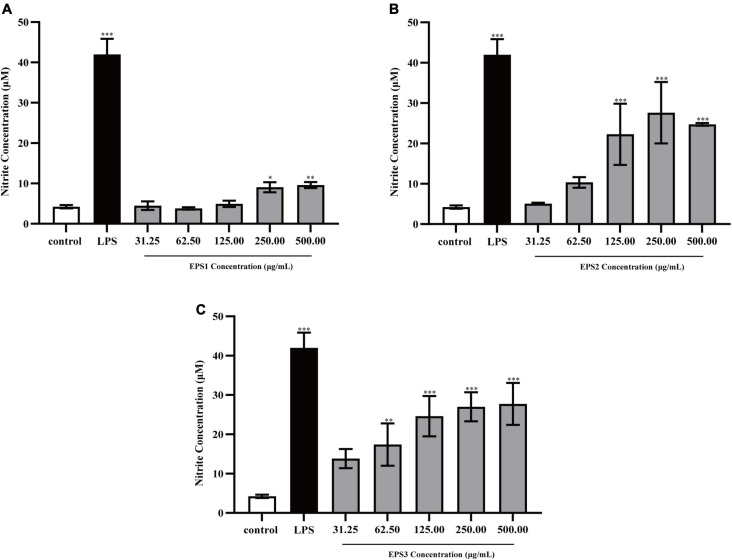
Production of NO from Ana-1 cells treated with **(A)** EPS1, **(B)** EPS2, and **(C)** EPS3 from *L. pantheris* TCP102. Data are expressed as the mean ± standard error (*n* = 6). *P < 0.05, ^**^P < 0.01, ^***^P < 0.001, compared to untreated control. Error bars represent ± SD.

**FIGURE 8:**
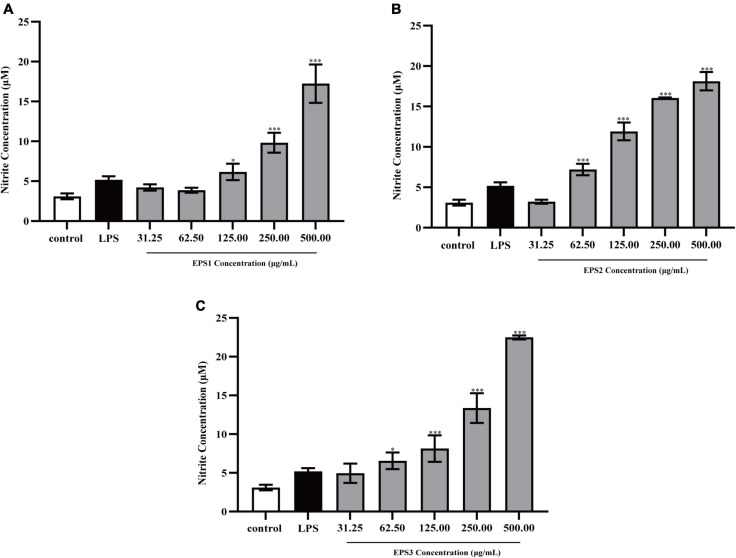
Production of NO from peritoneal macrophage cells treated with purified **(A)** EPS1, **(B)** EPS2, and **(C)** EPS3 from *L. pantheris* TCP102. Data are expressed as the mean ± standard error of the mean (*n* = 6). *P < 0.05, ^***^P < 0.001, compared to untreated control. Error bars represent ± SD.

Activated macrophages catalyze the generation of inducible NO synthase (iNOS), which results in the production of a large amount of NO from L-arginine and molecular oxygen (Alderton et al., 2001). NO is an important intracellular messenger molecule in living organisms as it is involved in killing microorganisms and tumor cells (Feng et al., 2002; Kleinert et al., 2003). Thus, we investigated the macrophage stimulating activities of the EPSs by measuring their ability to induce the release of NO from mouse macrophages. NO was quantified by measuring its stable breakdown product, nitrite. Because LPS significantly stimulates NO production, we included this as a positive control (Figure 7). EPS-induced NO production levels in Ana-1 and peritoneal macrophage cells were significantly increased in a dose-dependent manner. The results show significant differences among all concentrations of EPS (except for EPS1 at the concentration of 31.25 μg/mL), which suggested that these polysaccharides enhanced the ability of Ana-1 macrophages to secrete NO. EPS1 treatments did not differ significantly from untreated control (Figure 7A). However, EPS2 (125, 250, and 500 μg/mL) and EPS3 [125, 250, and 500 μg/mL (*P* < 0.001), 62.5 μg/mL (*P* < 0.01), 31.25 μg/mL (*P* < 0.05)] promoted NO production of macrophages considerably, when compared to the untreated cells (Figures 7B,C). The phagocytic effect of Ana-1 cells treated with higher concentrations of EPS (500 μg/mL) exhibited significant difference with that of positive control (*P* < 0.001). In addition, 62.5–500 μg/mL of all EPSs significantly increased the viability of peritoneal macrophage cells in a dose-dependent manner, compared to the blank control (*P* < 0.05) (Figure 8). Since the overproduction of NO can cause apoptosis in macrophages, the above results suggest that the macrophage activation of these three EPSs are more moderate than that of LPS.

#### Effect of exopolysaccharide on the production of cytokines

Cytokines are complex molecules that stimulate immune macrophages and play vital role in cell proliferation, intercellular interactions, and other functions (Kanamarlapudi and Muddada, 2017). IL-6 and TNF-α are the most important pro-inflammatory cytokines (Yuan et al., 2015). TNF-α induces the expression of many other immunoregulatory and inflammatory mediators and inhibits tumorigenesis. Meanwhile, IL-6 mediates the immune-enhancing activity by initiating the immunologic cascade during the induction of the acute phase protein response. In general, activated macrophages secrete cytokines, including TNF-α and IL-6 that are directly involved in inhibiting the growth of a wide range of invading substances as part of the immune-enhancing activity.

**FIGURE 9:**
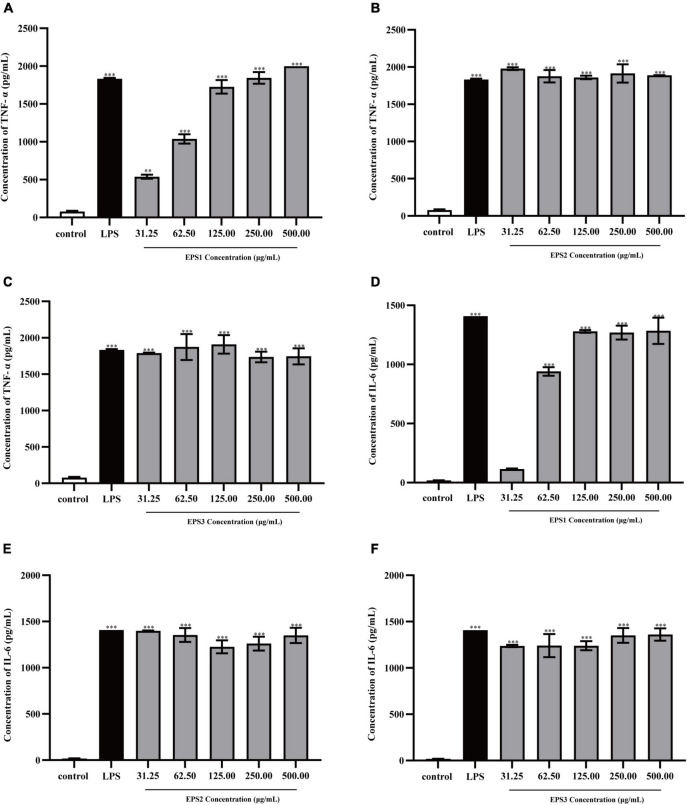
Production of the cytokines TNF-α by Ana-1 cells treated with purified **(A)** EPS1, **(B)** EPS2, and **(C)** EPS3. IL-6 production by Ana-1 cells treated with **(D)** EPS1, **(E)** EPS2, and **(F)** EPS3. Data are expressed as the mean ± standard error of the mean (*n* = 6). ^**^P < 0.01, ^***^P < 0.001, compared to untreated control. Error bars represent ± SD.

**FIGURE 10:**
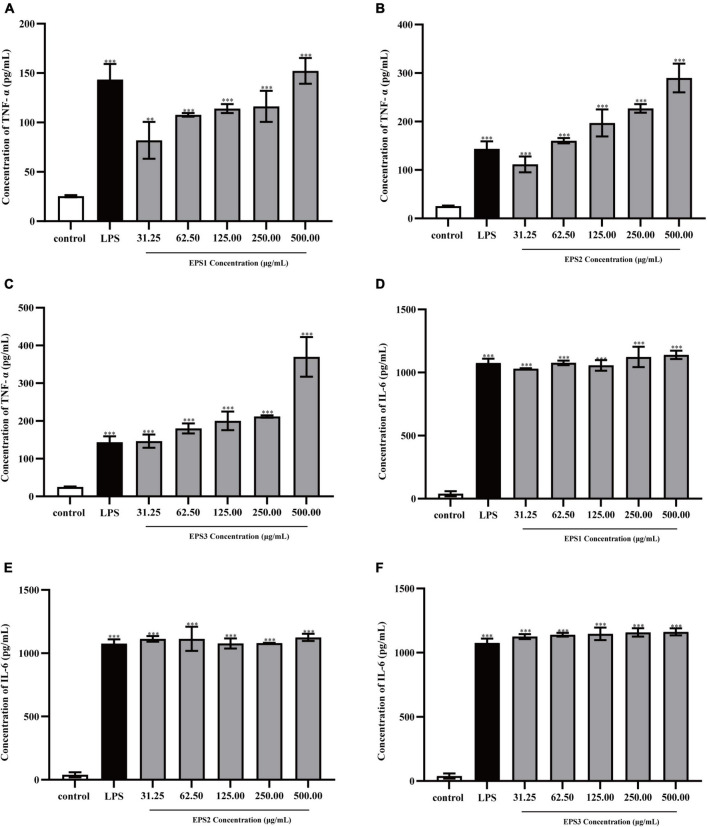
Production of the cytokines TNF-α by peritoneal macrophage cells treated with purified **(A)** EPS1, **(B)** EPS2, and **(C)** EPS3. Production of cytokine IL-6 by peritoneal macrophage cells treated with **(D)** EPS1, **(E)** EPS2, and **(F)** EPS3. Data are expressed as the mean ± standard error of the mean (*n* = 6). ^**^P < 0.01, ^***^P < 0.001, compared to untreated control. Error bars represent ± SD.

TNF-α and IL-6 levels in the culture supernatants of Ana-1 and peritoneal macrophage cells treated with purified EPSs were measured by ELISA (Figure 9). Various concentrations (31.25–500 μg/mL) of EPS1 (Figure 9A), EPS2 (Figure 9B), and EPS3 (Figure 9C) groups significantly (*P* < 0.001) increased the expression of TNF-α and IL-6 compared with the untreated cells, except for EPS1 at 31.25 and 62.5 μg/mL. Untreated cells secreted negligible amounts of these cytokines. In peritoneal macrophage cells treated with purified EPSs (Figure 10), all polysaccharides significantly induced the secretion of TNF-α (Figures 10A–C) and IL-6 (Figures 10D–F) in a dose-dependent manner. Compared with the control, all concentrations (from 31.25 to 500 μg/mL) of EPS1, EPS2, and EPS3 significantly increased the production of TNF-α and IL-6 (*P* < 0.05). These results demonstrate that EPSs secreted by *Lactobacillus pantheris* TCP102 could exert immune-enhancing activity by stimulating the release of TNF-α, IL-6 and NO in macrophages, which are essential for killing pathogens, microorganisms, and coordinating various biological activities as intracellular messenger molecules.

### Antitumor activity

Human colon cancer (HCT-116), ovarian cancer (A-2780), and gastric cancer (BCG-803) cell lines, are commonly used models in research on cancer cell proliferation.

**FIGURE 11:**
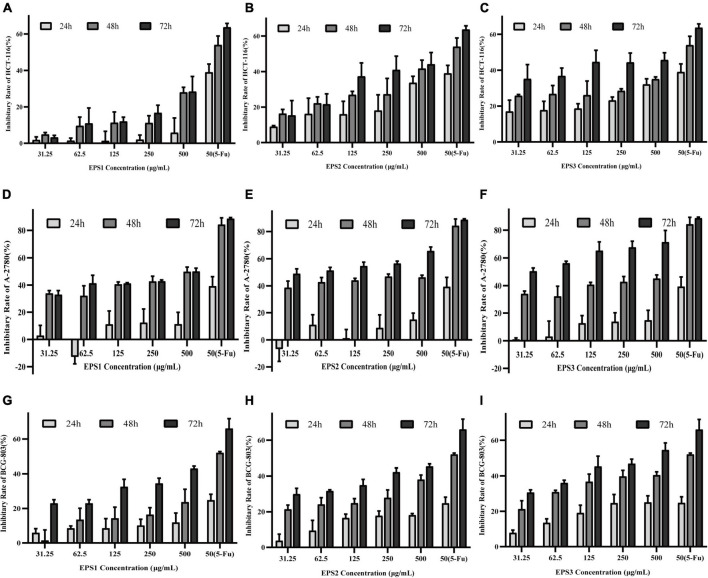
The effects of the purified EPS1, EPS2, and EPS3 from *L. pantheris* TCP102 and 5-fluorouracil on human colon cancer (HCT-116), ovarian cancer (A-2780), and gastric cancer (BCG-803) cell lines. Effect of **(A)** Effect of EPS1, **(B)** EPS2, and **(C)** EPS3 on HCT-116 at 24, 48, and 72 h treatment. Effect of **(D)** EPS1, **(E)** EPS2, and **(F)** EPS3 on A-2780 after 24, 48, and 72 h of treatment. Effect of **(G)** EPS1, **(H)** EPS2, and **(I)** EPS3 on BCG-803 after 24, 48, and 72 h of treatment. Data are expressed as the mean ± standard error of the mean (*n* = 6). Error bars represent ± SD.

Figure 11 shows that all three EPSs inhibit the proliferation of HCT-116, A-2780, and BCG-803 cells in concentration- and time-dependent manners. After 72 h of treatment, EPS3 exhibited the strongest antiproliferative activity against all cancer cell lines. At a dose of 500 μg/mL, HCT-116, A-2780, and BCG-803 cells were inhibited at rates of 45.68, 71.55, and 54.50%, respectively (Figures 11C,F,I). These results follow a similar as those of 5-FU (63.68, 88.77, and 66.04%). The antiproliferative effect of EPS2 against all three cancer cells was also time- and dosage-dependent (Figures 11B,E,H). After 72 h of incubation, HCT-116, A-2780, and BCG-803 were inhibited by 500 μg/mL of EPS2 at rates of 44.15, 56.61, and 45.33%, respectively. EPS1 inhibited the three tumor cells at rates lower than those of EPS2 and EPS3 under the same conditions (Figures 11A,D,G). Specifically, 500 μg/mL of EPS1 inhibited HCT-116, A-2780, and BCG-803 cells at rates of rates were 28.62, 50.17, and 43.05%, after 72 h of treatment. At concentrations of 31.25 and 62.5 μg/mL and 24 h of treatment, the antiproliferative activity of EPS1 ranged from low to no effected. Altogether, these results show that EPSs isolated from *L. pantheris* TCP102 have antitumor activities against HCT-116, A-2780 and BCG-803 cells ranging from moderate to strong (e.g., EPS3 against A-2780 cells). EPS3 has the strongest antiproliferative activity.

## Discussion

LAB strains such as *Lactobacilli* are the most common microorganisms which are considered as probiotic. There are several criteria for bacteria to recognize as probiotic. The main features for selecting highly potent probiotic species are their tolerance to acidic conditions and bile salts, ability to adhere to the intestinal cells, antibiotic resistance, survival in the GI-tract, and especially antimicrobial activity against pathogens (Mojgani et al., 2007).

In this work, the probiotic potential of 18 strains of lactic acid bacteria isolated from tomato pomace were evaluated by our laboratory, resistance to 0.3,0.5, and 1% bile salts, adherence to Caco-2 cells, and antagonism against some enteric pathogens. Our observations indicated that 11 isolated *Lactobacilli* (TCP001, TCP004, TCP007, TCP008, TCP009, TCP016, TCP063, TCP071, TCP073, TCP080, and TCP102) were resistant to gastric acid and 0.3, 0.5 and 1% bile salt, and exhibited good adhesion to Caco-2 cells. This study reveals that most isolates are more resistant to acidic condition than the control strain, which agrees with the previous report from Malaysia (Shokryazdan et al., 2014). In addition, the results show that these 18 strains have stronger bacteriostatic effect on *Staphylococcus aureus* than *Escherichia coli* which is in agreement with previous reports (Shokryazdan et al., 2014; Halimi and Mirsalehian, 2016). Antimicrobial activity is another criterion for selecting probiotic bacteria. Application of lactic acid bacteria as bio preservatives has been confirmed in various previous studies, due to their antagonistic effects against common foodborne bacteria (Jomehzadeh et al., 2020).

Recent studies show that various derived polysaccharides could stimulate NO and cytokines secretion in macrophages (Li et al., 2018), thereby stimulating the immune-enhancing activity. In this study, the three EPSs could exert immune-enhancing activity by stimulating the release of TNF-α, IL-6 and NO in Ana-1 and peritoneal macrophage cells. Previous studies show that the molecular structure, monosaccharide composition, chain conformation, and stereochemistry configuration of EPSs affect their immune-enhancing and anticancer activities (Ruas-Madiedo et al., 2002; Jung et al., 2015; Uzum et al., 2015). In the present study, we performed the first isolation and purification of EPS from the fermentation medium of *Lactobacillus pantheris* TCP102, resulting in three homogeneous exopolysaccharides, EPS1, EPS2, and EPS3. Purified EPS1, EPS2, and EPS3 have similar Mw, 20.3, 23.0, and 19.3 kDa, respectively, which are lighter than many other exopolysaccharides, such as EPS from *L. curvatus* DPPMA10, *L. plantarum* YW32, and *S. thermophiles* (Minervini et al., 2010; Wang et al., 2015; Ren et al., 2016). Polysaccharides with Mw between 10^4^ and 10^6^ Da, including galactose, glucose, and mannose, they are always related to immune-enhancing activity (Lee et al., 2018). Results from HPLC analysis indicate that EPS1, EPS2, and EPS3 are homogenized polysaccharides. EPS1, EPS2, and EPS3 are mainly composed of galactose, glucose, and mannose, at approximate molar ratios of 2.86:1:1.48, 1.26:1:1, 1.58:1.80:1, respectively. Therefore, the chemical composition and structure of the three EPSs were significantly beneficial for the immune-enhancing activity of polysaccharides. However, we only evaluate the immune-enhancing activity of EPSs on macrophages. Therefore, it is necessary to further probe the immune-enhancing activity mechanisms in the activation of macrophages by TCP102 EPS in the future. Furthermore, the EPSs isolated from *L. pantheris* TCP102 have antitumor activity against HCT-116, A-2780 and BCG-803 cells ranging from moderate to strong (e.g., EPS3 against A-2780 cells). EPS3 has the strongest antiproliferative activity. Other physicochemical properties of polysaccharides, such as the presence of uronic acid, pyranose, glucose, and β-type glycosidic linkages, are also conducive to increasing their anticancer activity (Ma et al., 2013). The differences in antitumor activities between them might be relating to their monosaccharide composition, Mw, and linkage types of glycosidic bond. The research on these factors affecting the immune-enhancing and antitumor activities of polysaccharides is not very in-depth, nor systematic, which needs further in-depth study. However, this study would provide scientific reference for further systematic investigation on the structure–bioactivity relationship of lactic acid bacteria polysaccharide.

## Conclusion

In conclusion, several isolates evaluated in this study showed potential probiotic properties, the majority of evaluated isolates showed high antimicrobial activities against potentially *Escherichia coli* and *Staphylococcus aureus*. In addition, the preparation, purification, structural characterization of polysaccharides, the immune-enhancing and antitumor activities of polysaccharides from *Lactobacillus pantheris* TCP102 were evaluated. The Mw of EPS1, EPS2 and EPS3 were estimated to be 20.3, 23.0, and 19.3 kDa, respectively. EPS1, EPS2 and EPS3 had the similar structure, and composed of galactose, glucose, and mannose, with molar ratios of 2.86:1:1.48, 1.26:1:1, 1.58:1.80:1, respectively. SEM results indicated the three polysaccharide fractions differ in microstructure and surface morphology. In addition, these EPSs significantly induced the production of nitric oxide (NO), TNF-α, and IL-6 in Ana-1 cells and peritoneal macrophage cells. Meanwhile, The EPSs also significantly suppressed the proliferation of HCT-116, BCG-803, and particularly A-2780 cells. Collectively, these findings indicate that EPSs from *Lactobacillus pantheris* TCP102 can be exploited as a potential immune-enhancing functional food and tumor-inhibiting drug.

## Data availability statement

The 16S-rRNA sequencing raw data are deposited in the online repositories. The names of the repository and accession number(s) can be found at: https://www.ncbi.nlm.nih.gov/, KF312693, KF312677–KF312692, and KF318727 for the strains TCP001, TCP007, TCP004, TCP008, TCP015–TCP017, TCP050, TCP029, TCP024, TCP045, TCP071, TCP073, TCP102, TCP080, TCP063, TCP037, and TCP009, respectively.

## Author contributions

SS and YF: conceptualization, investigation, formal analysis, and writing—original draft and preparation. NP, HZ, and LX: writing—review and editing. YLia, YLiu, BL, and CM: technical support. RD and XW: conceptualization, resources, supervision, funding acquisition, project administration, and writing—review and editing. All authors contributed to the article and approved the submitted version.
