# Supplementary material for: Novel exopolysaccharide derived from probiotic Lactobacillus pantheris TCP102 strain with immune-enhancing and anticancer activities
Source: Front Microbiol. 2022 Sep 26;13:1015270. doi: 10.3389/fmicb.2022.1015270 (PMC9549278; doi:10.3389/fmicb.2022.1015270)
Supplement: Supplementary file 1 [file Table_1.DOCX]

Supplementary Material

**Novel exopolysaccharide derived from probiotic *Lactobacillus pantheris* TCP102 strain with immune-enhancing and anticancer activities**


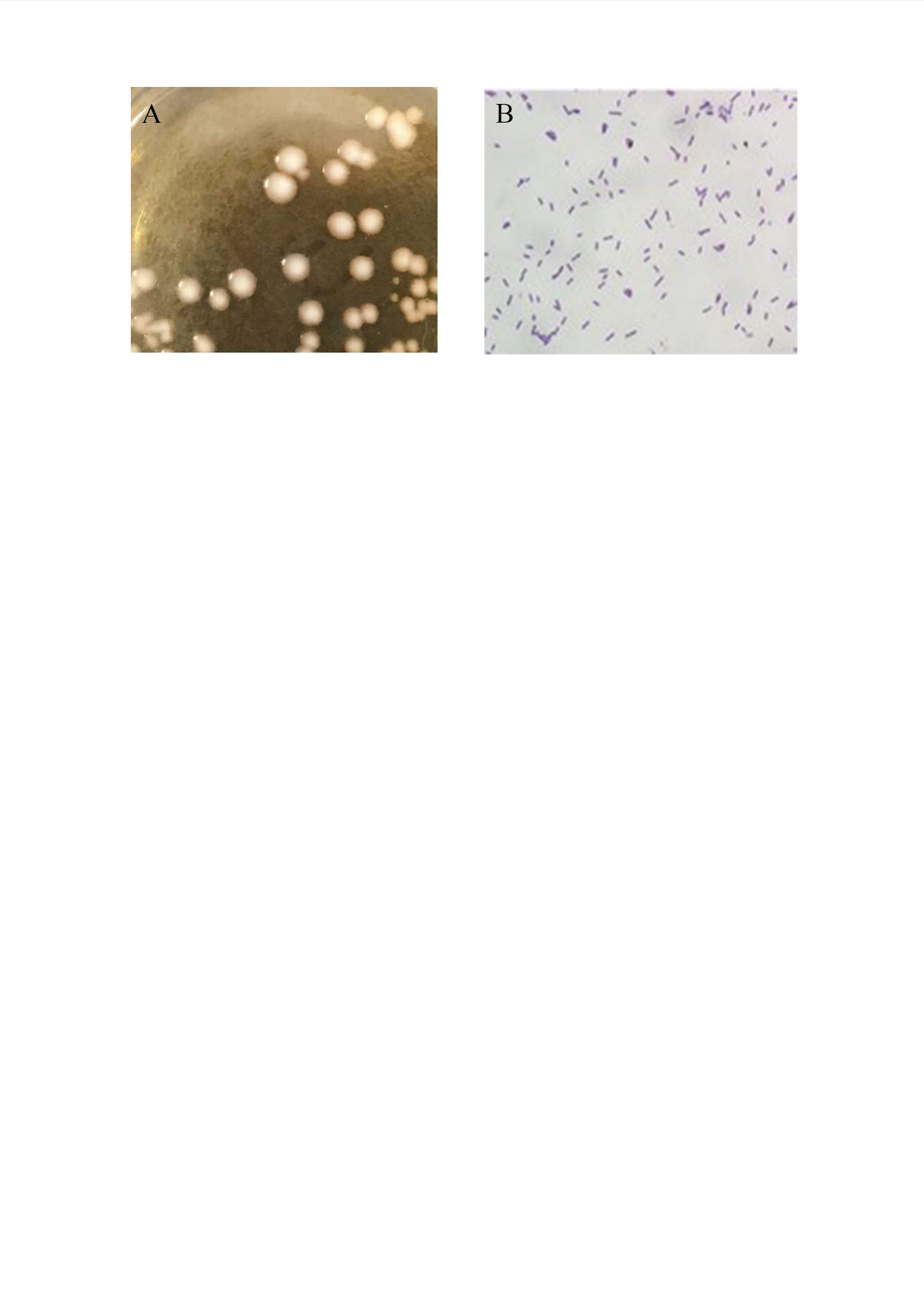


**Supplementary Figure S1.** Identification of strain TCP102: (A) the colony morphology; (B) micrograph (100× magnification).
